# Oolong tea cultivars categorization and germination period classification based on multispectral information

**DOI:** 10.3389/fpls.2023.1251418

**Published:** 2023-08-29

**Authors:** Qiong Cao, Chunjiang Zhao, Bingnan Bai, Jie Cai, Longyue Chen, Fan Wang, Bo Xu, Dandan Duan, Ping Jiang, Xiangyu Meng, Guijun Yang

**Affiliations:** ^1^ Technology Research Center, Beijing Academy of Agriculture and Forestry Sciences, Beijing, China; ^2^ Hunan Agricultural University College of Mechanical and Electronical Engineering, Changsha, Hunan, China

**Keywords:** oolong tea cultivar, multispectral characteristics, SVM, identification, and germination

## Abstract

Recognizing and identifying tea plant (*Camellia sinensis*) cultivar plays a significant role in tea planting and germplasm resource management, particularly for oolong tea. There is a wide range of high-quality oolong tea with diverse varieties of tea plants that are suitable for oolong tea production. The conventional method for identifying and confirming tea cultivars involves visual assessment. Machine learning and computer vision-based automatic classification methods offer efficient and non-invasive alternatives for rapid categorization. Despite advancements in technology, the identification and classification of tea cultivars still pose a complex challenge. This paper utilized machine learning approaches for classifying 18 oolong tea cultivars based on 27 multispectral characteristics. Then the SVM classification model was executed using three optimization algorithms, namely genetic algorithm (GA), particle swarm optimization (PSO), and grey wolf optimizer (GWO). The results revealed that the SVM model optimized by GWO achieved the best performance, with an average discrimination rate of 99.91%, 93.30% and 92.63% for the training set, test set and validation set, respectively. In addition, based on the multispectral information (h, s, r, b, L, Asm, Var, Hom, Dis, σ, S, G, RVI, DVI, VOG), the germination period of oolong tea cultivars can be completely evaluated by Fisher discriminant analysis. The study indicated that the practical protection of tea plants through automated and precise classification of oolong tea cultivars and germination periods is feasible by utilizing multispectral imaging system.

## 1 Introduction

In most nations, tea plant (*Camellia sinensis*) is a significant economic crop, with potential medicinal application (Pan et al., 2022). Most tea plantation management activities are conducted without the utilization of intelligent technology. The procedure of growing and producing tea heavily depends on skilled human workers, resulting in a labor-intensive and inefficient process (Zhang et al., 2023). Identifying tea cultivars plays a crucial role in the tea industry as it directly impacts the commercial tea’s yield and quality. There is great variation in taste and quality among different cultivars of the tea plant (*Camellia sinensis*) (Yue et al., 2023; Zaman et al., 2022). Improving the identification and assessment of tea cultivars is essential in tea processing to ensure the production of tea with high quality and yield. The identification of tea plant cultivars is typically reliant on human observation, and it requires expert judgement and time to manually determine the cultivar type. It becomes challenging to differentiate between a series of tea plant cultivars when they appear highly similar, making manual identification difficult. Biochemical and molecular methods used for laboratory analysis are expensive, complex, and can cause damage to the sample. Oolong tea is a famous Chinese tea category, which quality is significantly affected by the cultivar of tea plant (Lin et al., 2022). Most oolong tea products are named after their cultivar names, like Benshan, Dahongpao, Tieluohan. Accurate identification and classification of oolong tea cultivar contribute to the protection and preservation of different genetic resources, preventing the loss and reduction of genetic diversity. Cultivar identification enable targeted quality control measures to ensure oolong tea product consistency and quality stability. Oolong tea cultivars identification provides guidance for cultivation management, including suitable growing environments, fertilization, and pest control measures. Therefore, there is an urgent need for a fast and non-invasive method to identify oolong tea plant cultivars.

The extensive use of remote sensing techniques for plant monitoring in the field has made it feasible to quickly identify tea cultivars. While it is possible to classify various types of vegetation using remotely sensed images, the identification of different cultivars is relatively uncommon. Several studies have differentiated between tea plantations and extract tea plantations using multispectral satellite image, as demonstrated by Chen et al. (Chen et al., 2022) and Zhu et al. (Zhu et al., 2019). Bao et al. (Bao et al., 2023) utilized a UAV platform to detect tea leaf blight, while Tu et al. (Tu et al., 2018) employed hyperspectral information obtained from a UAV to classify tea plant cultivars. The use of thermal images holds promise for applications in plant research. For example, thermal cameras were utilized by Batchuluun et al. (Batchuluun et al., 2022) for the purpose of classifying plants and identifying diseases. Furthermore, the integration of spectral information and image information through spectral imaging analysis, such as hyperspectral (Zhao et al., 2022), near-infrared spectral, and multispectral, has demonstrated significant benefits in nondestructive detection, identification, quality evaluation, and safety control of agricultural products. Zou et al. (Zou et al., 2023) evaluated Mengding mountain green tea varieties using hyperspectral image. Wang et al. (Wang et al., 2021) applied near-infrared hyperspectral imaging to analyze spatial distribution of total polyphenols in tea. Cao et al. (Cao et al., 2022a) combined hyperspectral and multispectral information to monitor tea plant growth.

Typical spectral images obtained through multispectral imaging can also offer plentiful information regarding the object of detection. Multispectral imaging (MSI) technology has been demonstrated in numerous studies to allow for non-invasive and unbiased identification of plant phenotyping (Chen et al., 2021), including but not limited to assessing fruit quality (Liu et al., 2015) and distinguishing between different crop varieties (Liu et al., 2016). By utilizing MSI technology, Cao et al. (Cao et al., 2022b) successfully developed a model for distinguishing 16 different types of tea cultivars, achieving highly accurate classification results. Th e use of multispectral imaging in conjunction with machine learning algorithms in these applications has established a foundation of knowledge and expertise for utilizing MSI technology to accurately identify tea cultivars. Currently, there are few studies on techniques for on-site identification of specific tea varieties, particularly for oolong tea varieties. Therefore, it is urgent to investigate a quick and efficient alternative to the traditional laborious and subjective methods used for categorizing oolong tea cultivars. The aim of this study was to utilize MSI to enhance the identification of tea cultivars for effective tea plant management and to facilitate phenotyping for cultivars with high yield. In this study, multispectral images of the canopies of different oolong tea cultivars were captured using a multispectral camera (RedEdge-MX, Micasense, Seattle, WA, USA). Then, oolong tea cultivars were classified based on the color indicators, spectral characteristics, and texture features of the tea canopies.

## 2 Data and methods

### 2.1 Data acquisition

**Figure 1:**
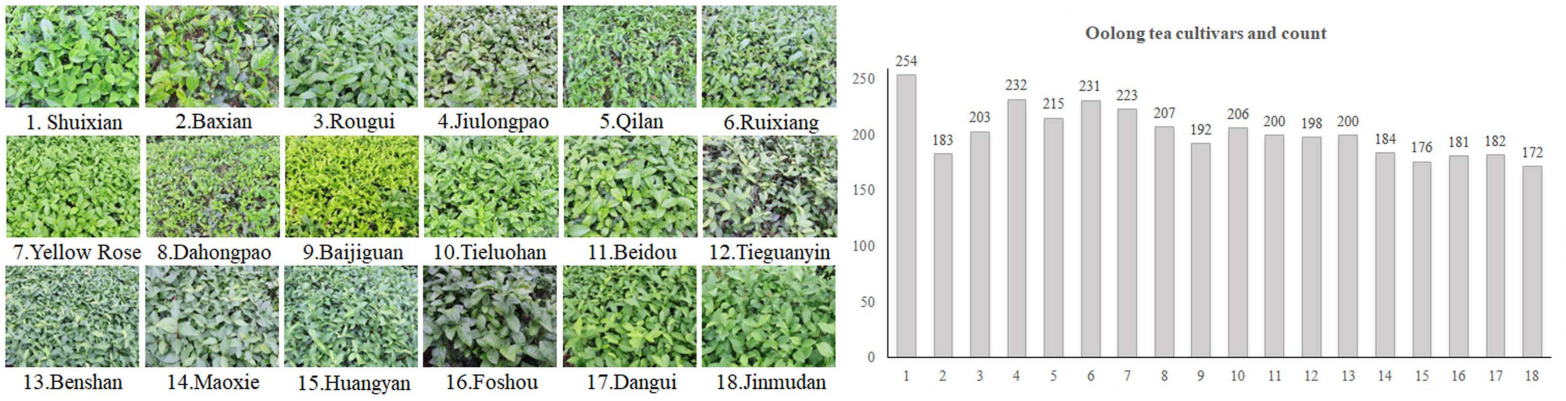
Oolong tea cultivars and count.

The experiment was done in a tea farm located in Anxi County, Quanzhou City, Fujian Province, China. Fujian is one of the major producing areas of oolong tea. Anxi has been producing tea for thousands of years and is renowned as the birthplace of Anxi Tieguanyin and “the hometown of Chinese Oolong Tea (Famous Tea)”. It is considered the top tea-producing county in China and is widely recognized as “China’s Tea Capital”. The research site possesses a marine monsoon climate of mid-subtropical region in the southern hemisphere, with an average yearly temperature ranging from 19 to 21°C and an annual precipitation of 1600 mm, which renders it an appropriate environment for the growth of tea plants. **Figure 1** displays information regarding the oolong tea cultivars examined in this experiment.

The examined set consisted of 18 types of plant cultivars that are suitable for producing oolong tea. These cultivars originated in Fujian, and were subsequently introduced to Guangdong, Zhejiang, and Jiangnan tea regions. Based on their germination period, they can be divided into three stages: early species (Baxian, Yellow Rose, Huangyan, Dangui, Jinmudan), mesophytic species (Baijiguan, Beidou, Benshan, Maoxie, Foshou), late species (Shuixian, Rougui, Jiulongpao, Qilan, Ruixiang, Dahongpao, Tieluohan, Tieguanyin).

In this experiment, a multispectral camera with five bands, including blue (B), green (G), red (R), near infrared (NIR), and red edge (RED) was used. The center wavelengths of these bands were 475nm, 560nm, 668nm, 840nm, and 717nm. The images were captured on a sunny day between 10:00 am and 3:00 pm to minimize the impacts of illumination changes. The camera was positioned 120cm above tea plant canopy. A barium sulfate (BaSO_4_) reference board was placed on the tea plant canopy to calibrate the spectral reflectance. The calibration equation was calculated using:


(1) $$R_{i}=\frac{DN_{i}*R_{s}}{DN_{s}}$$

where, $DN_{s}$ and $DN_{i}$ represented the digital number of the stand reference board and multispectral images, respectively, and $R_{s}$ and $R_{i}$ indicated the reflectance of the standard reference board and multispectral images, respectively.

Over the course of two years, in May 2020 and October 2021, a total of 3639 multispectral images were captured for 18 different oolong tea cultivars.

### 2.2 Multispectral data processing

The initial stage of processing multispectral images involves the registration of images and fusion of bands to correct for any spatial deviation or misalignment that may have occurred during image acquisition due to sensor layout and movement. The SIFT algorithm has proven to be effective in both multispectral image band fusion and information sampling, as it facilitates the automatic selection and matching of feature points within each band image. To eliminate the impact of soil, shadows, and other backgrounds, the raw images were used to isolate the fresh leaves of the tea plant canopy. Previous research has shown that ExGR is effective in extracting plant and crop images. The ExGR value was determined using the following formula:


(2) ExGR=(2*G−R−B)−(1.4*R−G)

The ExGR can be utilized to improve the distinctive features of tea leaves in multispectral images of the tea plant canopy and enhance their contrast with other objects on the ground, facilitating the differentiation of tea leaves from the background. The Otsu method was employed to segment the images, followed by background masking to isolate the tea leaves of the tea plant canopy. For a comprehensive understanding of the multispectral image processing procedures, please refer to Cao et al. (Cao et al., 2022b).

### 2.3 Multispectral feature extraction

The multispectral images provide abundant information that can be analyzed. Color, texture, and spectral behaviors are the key monitoring indicators of tea plant growth and markedly differ among tea cultivars. For this study, data on color values, texture, and reflectance were obtained from five different bands of tea canopy images. Additionally, various color and vegetation indices were calculated as the primary characteristics. A sum of 86 indicators were chosen for this study, encompassing color value, color indicators, texture data, single band reflectance, and vegetation indices.

#### 2.3.1 Color information

For describing color information, we have selected three color models: RGB, HSV, and CIE L*a*b*. We obtained basic color indicators for each sample by calculating the mean of 9 color values (r, g, b, h, s, v, L*, a*, b*) from masked images. Furthermore, advanced color indicators were derived by computing 7 color indices., as listed in **Table 1**.

**Table 1:** Color indicators complied from the literature.

CIs	Formula	Reference	CIs	Formula	Reference
LI	L*-b*	(Wahono et al., 2019)	AI	b*-a*	(Wahono et al., 2019)
AL	a*/L*	(Wahono et al., 2019)	AB	a*/(L*-b*)	(Wahono et al., 2019)
NDLBI	(L*-b*)/(L*+b*)	(Wahono et al., 2019)	NDALI	(a*-L*)/(a*+L*)	(Wahono et al., 2019)
NDABI	(a*-b*)/(a*+b*)	(Wahono et al., 2019)			

#### 2.3.2 Texture information

To quantitatively describe the texture features of the tea plant canopy and differentiate among the 18 oolong tea cultivars, texture analysis methods such as grey-level co-occurrence matrix (GLCM) and local binary patterns (LBP) were utilized. The GLCM was proposed by Haralick et al. (Haralick et al., 1973) in 1970s. The principle of GLCM involves analyzing the relationship between the gray values of pixels in a grayscale image. This is achieved by calculating the probability (Pij) of a particular gray value occurring in a fixed pixel, alongside the gray value of another pixel located at a certain distance (d) and direction (θ) within the image area. The values of θ include 0°, 45°, 90°, and 135°. By analyzing these relationships, GLCM can provide useful statistical information about the texture of an image, such as contrast, homogeneity, and entropy. The LBP operator is frequently employed to characterize the texture properties of grayscale images due to its ability to maintain gray and rotational invariance. The principle of LBP operator is to compare the intensity values of a central pixel with its surrounding pixels in a circular neighborhood. The pixels are then classified as 1 or 0 based on whether their intensity values are greater than or less than that of the central pixel. This process generates a binary code that represents the local texture of the image. The LBP operator has the advantage of being able to maintain gray invariance, which means that it is not affected by changes in illumination. It is also rotation invariant, meaning that the texture features can be extracted regardless of the orientation of the image. This texture information can then be used for image processing tasks such as feature extraction, segmentation, and classification. This study involved the calculation of 8 features using GLCM and 7 features using LBP, as shown in **Table 2**.

**Table 2:** Texture indices (TI) calculated by GLCM and LBP.

TI	Formula	Method		TI	Formula	Method	
Asm	$\sum_{\text{i}}\sum_{\text{j}}\text{P}{\left(\text{i},\text{j}\right)}^{2}$	GLCM	(3)	μ	$\sum_{g=0}^{L-1}gP\left(g\right)$	LBP	(11)
Ent	$-\sum_{\text{i}}\sum_{\text{j}}\text{p}\left(\text{i},\text{j}\right)\text{log}\left(\text{p}\left(\text{i},\text{j}\right)\right)$	GLCM	(4)	σ	$\sqrt{\sum_{g=0}^{L-1}{\left(g-\mu \right)}^{2}P\left(g\right)}$	LBP	(12)
Con	$\sum_{\text{n}=0}^{{\text{N}}_{\text{g}}-1}{\text{n}}^{2}{\{\sum_{\text{i}=1}^{{\text{N}}_{\text{g}}}\sum_{\text{j}=1}^{{\text{N}}_{\text{g}}}\text{p}\left(\text{i},\text{j}\right)\}}_{\left|\text{i}-\text{j}\right|=\text{n}}$	GLCM	(5)	S	$\sum_{g=0}^{L-1}{\left(g-\mu \right)}^{3}P\left(g\right)$	LBP	(13)
Cor	$\frac{\sum_{\text{i}}\sum_{\text{j}}\left(\text{ij}\right)\text{p}\left(\text{i},\text{j}\right)-{\text{μ}}_{\text{x}}{\text{μ}}_{\text{y}}}{{\text{σ}}_{\text{x}}{\text{σ}}_{\text{y}}}$	GLCM	(6)	K	$\frac{1}{\sigma^{4}}\sum_{g=0}^{L-1}{\left(g-\mu \right)}^{4}P\left(g\right)$	LBP	(14)
Mea	$\sum_{\text{i},\text{j}=0}^{\text{N}-1}\text{i}\left({\text{P}}_{\text{i},\text{j}}\right)$	GLCM	(7)	G	$\sum_{g=0}^{L-1}P{\left(g\right)}^{2}$	LBP	(15)
Var	${\text{σ}}_{i}^{2}=\sum_{\text{i},\text{j}=0}^{\text{N}-1}{\text{P}}_{\text{ij}}{\left(\text{i}-{\text{μ}}_{\text{i}}\right)}^{2}\;$ ${\text{ σ}}_{\text{j}}^{2}=\sum_{\text{i},\text{j}=0}^{\text{N}-1}{\text{P}}_{\text{ij}}{\left(\text{j}-{\text{μ}}_{\text{j}}\right)}^{2}$	GLCM	(8)	E	$-\sum_{g=0}^{L-1}P\left(g\right)\log_{2}\left[P\left(g\right)\right]$	LBP	(16)
Hom	$\sum_{\text{i},\text{j}=0}^{\text{N}-1}\frac{{\text{P}}_{\text{i},\text{j}}}{1+{\left(\text{i}-\text{j}\right)}^{2}}$	GLCM	(9)	R	$\frac{1}{1+\sigma^{2}}$	LBP	(17)
Dis	$\sum_{\text{i},\text{j}=0}^{\text{N}-1}{\text{P}}_{\text{i},\text{j}}\left|\text{i}-\text{j}\right|$	GLCM	(10)				

#### 2.3.3 Spectral information

The pigments present in plants mainly influence their reflectance in the visible range, while the reflectance in the NIR range is primarily determined by the cellular structure and canopy morphology (Yuan et al., 2019). The reflectance values in the Red and NIR bands provide valuable information that can be used to assess the biophysical condition of plants. For the original dataset, we utilized both the reflectance values of individual bands and various vegetation indices. We evaluated a set of 50 traditional vegetation indices that are linked to plant pigments, water levels, plant stress, and other biochemical characteristics including cellulose and lignin. **Table 3** lists the formula for calculations.

**Table 3:** Vegetation indices complied from the literature.

VIs	Formula	Reference	VIs	Formula	Reference
NDVI	(NIR-R)/(NIR+R)	(Main et al., 2011)	RVI	NIR/R	(ELeblanc, 2000)
DVI	NIR-R	(ELeblanc, 2000)	EVI	2.5(B-G)/(B+6G-7.5R+1)	(Ahamed et al., 2011)
VOG	(B-G)/(R+RED)	(VOGELMANN et al., 1993)	MTCI	(B-G)/(R-RED)	(Main et al., 2011)
GNDVI	(NIR-G)/(NIR+G)	(Merzlyak, 1996)	RDVI	(NIR-RED)/ $\sqrt{\left(NIR+RED\right)}$	(ELeblanc, 2000)
OSAVI	1.16(NIR-RED)/(NIR+RED+0.16)	(Kramer, 2001)	NLI	(NIR^2^-RED)/(NIR2+RED)	(Kramer, 2001)
TGI	G-0.39R-0.61B	(Bannari et al., 2011)	ExG	2G-R-B	(Mortensen, 1995)
VARI	(R-G)/(G+R-B)	(Merzlyak et al., 1999)	NDRE	(NIR-RED)/(NIR+RED)	(Merzlyak et al., 1999)
WDRVI	(0.1NIR-R)/(0.1NIR+R)	(Ahamed et al., 2011)	GRVI	(G-R)/(G+R)	(Ahamed et al., 2011)
PSRI	(R-G)/RED	(Merzlyak et al., 1999)	PGR	R/G	(Haboudane et al., 2004)
CCCI	((NIR-RED)/(NIR+RED))/((NIR-R)/(NIR+R))	(Haboudane et al., 2004)	MCARI	((RED-R-0.2(RED-G))*(RED/R)	(Haboudane et al., 2004)
BGI	B/G	(Frutos, 2005)	BI	(NIR+R+G)/ √3	(Frutos, 2005)
GI	G/R	(Frutos, 2005)	SIPI	(NIR-B)/(NIR+R)	(Main et al., 2011)
PVI	(1/ $\sqrt{1.17^{2}+1}\;$ )(NIR-1.17R-3.37)	(ELeblanc, 2000)	SAVI	(NIR-R)/(NIR+R+0.5)*1.5	(Ahamed et al., 2011)
SR	NIR/R	(Ahamed et al., 2011)	GDVI	NIR-G	(Merzlyak et al., 1999)
RI	RED/R	(Kramer, 2001)	RGI	R/G	(Ceccato et al., 2001)
BRI	B/R	(Major et al., 1990)	GMR	G-R	(Wang et al., 2013)
NRI	R/(R+G+B)	(Ansari et al., 2021)	NGI	G/(R+G+B)	(Ansari et al., 2021)
INT	(R+G+B)/3	(Kawashima and N., 1998)	NBI	B/(R+G+B)	(Ansari et al., 2021)
NDI	128(G-R)/(R+G)	(Woebbecke et al., 1993)	WI	(G-B)/(|R-G|)	(Mortensen, 1995)
ExR	1.4*R-G	(Castillo-Martinez et al., 2020)	ExGR	ExG –ExR	(Castillo-Martinez et al., 2020)
CIVE	0.441R-0.811G+0.385B+18.78745	(Castillo-Martinez et al., 2020)	NGRDI	(G-R)/(G+R)	(Castillo-Martinez et al., 2020)
VEG	G/R^2/3^*B^1/3^	(Hague et al., 2006)	COM1	ExG+CIVE+ExGR+VEG	(Lu et al., 2022)
COM2	0.36ExG+0.47CIVE+0.17VEG	(Lu et al., 2022)	RGBVI	(G^2^-R*B)/(G^2^+R*B)	(Bendig et al., 2015)
MGRVI	(G^2^-R^2^)/(G^2^+R^2^)	(Bendig et al., 2015)	MExG	1.262G-0.884R-0.311B	(Burgos-Artizzu et al., 2011)
NDYI	(G-B)/(G+B)	(Sulik and Long, 2016)	GLI	(2G-R-B)/(2G+R+B)	(Louhaichi et al., 2001)

### 2.4 Feature selection

The primary purpose of feature screening is to decrease the data dimensionality, simplify post-processing, and eliminate irrelevant or incorrect information that may affect the final classification outcomes. Due to the high dimensionality of the extracted data, feature selection was performed prior to modeling. The feature selection technique was utilized to identify a subset of indicators that had minimal collinearity, negligible redundancy, and valuable information to effectively represent all the multispectral data.

Uninformation variable elimination (UVE) has a notable advantage in selecting wavelengths, as it combines both noise and spectral information to extract the characteristic wavelengths of the spectrum. The outcomes of UVE’s selection process are more straightforward and easier to interpret. The UVE approach utilizes the partial least squares regression coefficient as the primary criterion for wavelength selection (Wang et al., 2020), enabling the identification of valuable wavelengths while removing irrelevant or redundant data. This method ensures that only useful wavelengths are retained for further analysis. Wang et al. (Wang et al., 2022) utilized the UVE method to enhance the predictive accuracy of the nitrogen and carbon content assessment model for maize canopy by utilizing NIR spectra. Shen et al. (Shen et al., 2022) applied UVE algorithm to select variables from THz spectra, for determining the origin of wheat. Previous research has shown that the parameters obtained by UVE method of the prediction mode are superior to the full-spectrum modelling model.

Least Absolute Shrinkage and Selection Operator (LASSO) is a shrinkage estimation algorithm proposed by Tibshirani (Robert, 1996). It is a regression analysis method that performs both variable selection and regularization to improve the accuracy and interpretability of the model. The principle of LASSO is to minimize the sum of the squared errors between the predicted values and the actual values, subject to a constraint that the sum of the absolute values of the coefficients is less than or equal to a specified constant. This constraint forces some of the coefficients to be exactly zero, effectively performing variable selection and removing irrelevant or redundant features from the model. The LASSO algorithm uses λ to control the strength of the constraint and balance the trade-off between model complexity and predictive accuracy. By adjusting the value of λ, the LASSO algorithm can produce a sequence of models with different numbers of non-zero coefficients. The use of LASSO variable selection has become more popular in the fields of bioinformatics and stoichiometry (Massaro et al., 2023). Based on the advantages of UVE and LASSO algorithms, this paper introduced them into the feature screening of tea canopy spectral image information.

### 2.5 Classification models of oolong tea cultivar

Previous research has suggested that the SVM algorithm exhibits certain benefits in categorizing different types of tea plants, achieving a relatively high level of precision (Cao et al., 2022b). The SVM algorithm was utilized as the primary classification technique for identifying oolong tea cultivars in this study. One major benefit of the SVM algorithm as a supervised classification approach is that it does not necessitate prior knowledge. The fundamental idea of SVM learning is to determine the separation hyperplane that can effectively divide the training dataset with the greatest margin (Phillips and Abdulla, 2021). This hyperplane is selected to have the largest possible margin, representing the distance between the hyperplane and the nearest data points from each class. By maximizing the margin, SVM aims to achieve improved generalization performance and robustness. The penalty factor “c” and kernel parameter “g” have a great influence on the performance of SVM in tea cultivars classification, directly affecting the accuracy and reliability of classifier model. The optimization problem of SVM involves finding the optimal hyperplane parameters that minimize the classification error while maximizing the margin. This is formulated as a convex optimization problem, which can be solved efficiently using optimization algorithms. Therefore, it is necessary to find an intelligent algorithm that can be employed for optimizing SVM parameters to improve classification accuracy. The current study utilizes and explores GA, PSO, and GWO algorithms to acquire the optimal c and g values, thereby enhancing the accuracy of SVM-based tea cultivar identification.

Genetic algorithm (GA) is a powerful optimization technique inspired by biological evolution and natural selection, which employs adaptive probabilities to search for the optimal solution globally. Initially introduced by Professor Holland as a heuristic search method based on Darwin’s theory of evolution, GA produces the succeeding generation of solutions using heredity operations such as selection, crossover, and mutation. This process ensures that only individuals with high fitness function values are preserved, while those with low values are gradually eliminated. By repeating this cycle, optimal solutions that meet the specified constraints can be obtained, enabling optimization objectives to be achieved. GA has global search capabilities. It effectively searches the parameter space of SVM through genetic operations, making it suitable for fine-tuning SVM model parameters.

Particle swarm optimization (PSO) is derived from the behavior of bird predation patterns. In PSO, each bird is represented as a random particle, characterized by its position and speed. The particle’s initial position and speed are randomly assigned, and through iterative updates, the particle moves towards an optimal solution. During each iteration, the algorithm generates the global extreme value, which is the optimal solution across the entire population, as well as the individual extreme value, which is the optimal solution for each individual particle. PSO has both global and local search capabilities. It effectively addresses the parameter optimization of SVM by updating particle positions and velocities to explore the parameter space and find the optimal solution. PSO converges quickly and produces good results for SVM parameter optimization. The detail algorithm described SVM optimized by PSO can be referenced in Liu et al. (Liu et al., 2019).

Grey Wolf Optimization Algorithm (GWO) is a nature-inspired algorithm that mimics the hunting behavior of wolves to search for optimal prey. The hunting process involves three main stages: tracking, encircling, and attacking. In the context of parameter optimization, GWO can efficiently search for the optimal penalty parameter c and kernel parameter g for tea tree variety classification model system, thereby achieving the objective of parameter optimization. GWO algorithm demonstrates good convergence and speed and strong global search capabilities. It effectively addresses the parameter optimization of SVM by updating parameter values based on the search behavior of grey wolves, aiming to find the optimal solution, yielding favorable results for SVM parameter optimization. It has been found to be a promising technique for improving accuracy in classification problems by optimizing SVM parameters by GWO. (Barman and Choudhury, 2020).

The datasets were split into training, testing, and validation sets in a 6:2:2 ratio, resulting in a total of 3639 datasets. The model’s accuracy was determined by calculating the ratio of correct samples to total samples for each tea cultivar. All data analysis was performed using Matlab 2017b ((MathWorks Inc., Natick, MA, USA)).

## 3 Results

### 3.1 Characteristic indicators analysis

**Figure 2:**
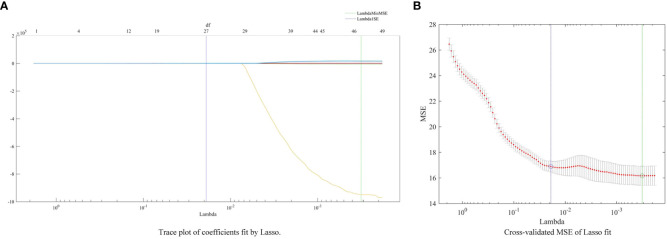
The process of indicators selected by LASSO.

Initially, a total of 86 characteristics were extracted as input data. However, after applying the UVE algorithm, only 57 indicators remained. Subsequently, the LASSO algorithm was employed following UVE. In the first step, cross-validation was used to select the optimal λ for the model. This ensured minimal fluctuation and stability of the cross-validation error. Ultimately, 27 indicators were retained after UVE-LASSO algorithms. These indicators are h, s, r, b, L*, Asm, Var, Hom, Dis, σ, S, G, RVI, DVI, VOG, MTCI, NLI, VARI, MCARI, BGI, SR, RI, GMR, ExR, AL, NDLBI, and NDALI. **Figure 2** depicts the process of indicators selected by LASSO.

### 3.2 Classification results based on different parameter optimization algorithms

**Figure 3:**
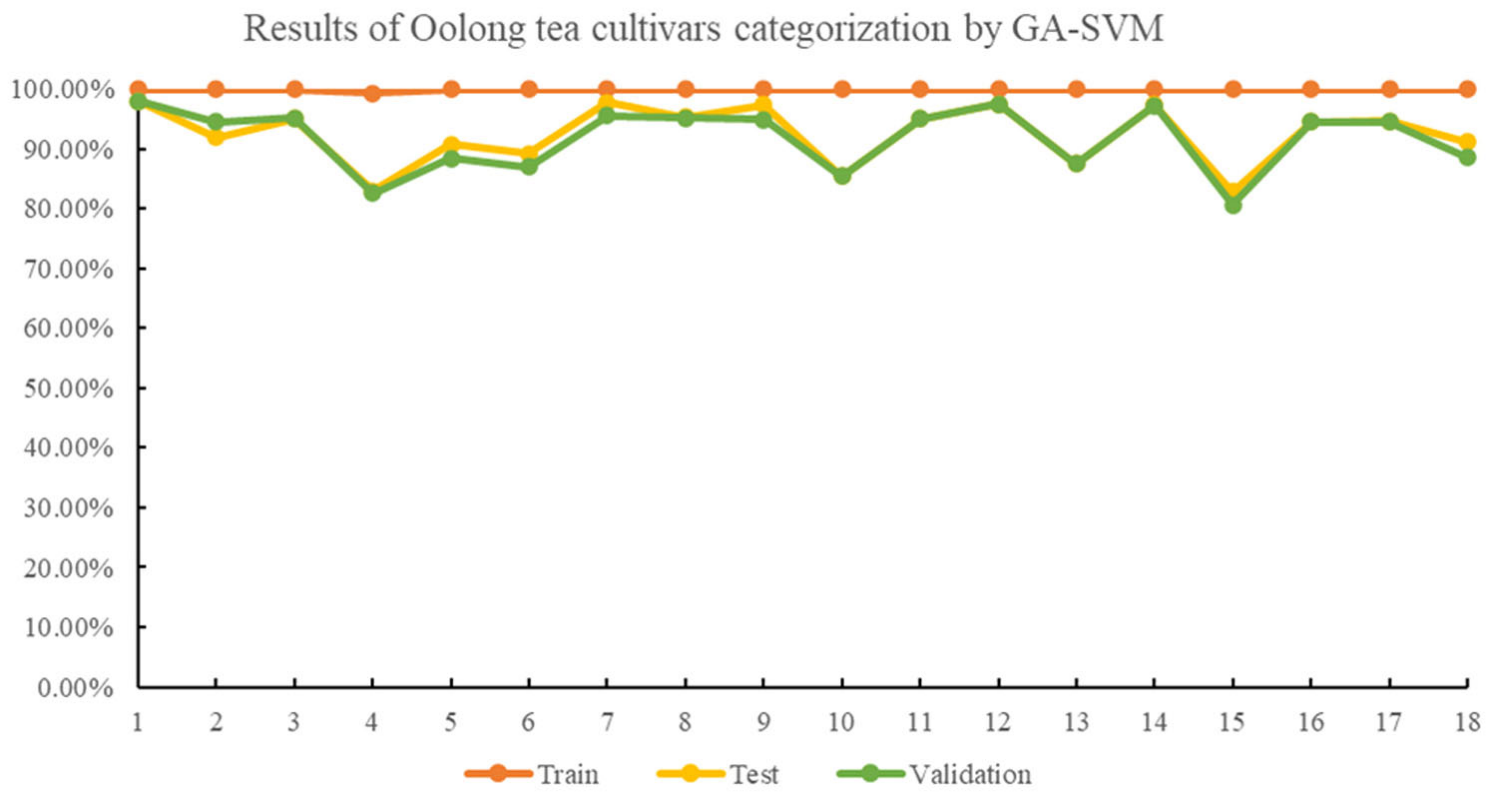
Results of oolong tea cultivar categorization by GA-SVM.

The multispectral imaging system has shown to be a valid method for qualitatively and quantitatively monitoring tea quality (Chen and Yan, 2020; Chen et al., 2021), and can assist in identifying tea plant varieties using the SVM method (Cao et al., 2022b). Initially, the SVM algorithm was applied to classify oolong tea cultivars, and achieved average accuracies of 99.79%, 91.31% and 90.62% for the training, test, and validation sets. However, the identification rate for the Huangyan cultivar was less than 80%. To improve the efficiency of oolong tea cultivars, optimized algorithms, including GA, PSO, and GWO, were proposed to optimize the parameters c and g of the SVM model. In the GA-SVM model, satisfactory results were obtained with best c and g values of 31.2540 and 8.3673, respectively, and the average accuracies for the training, testing, and validation sets were 99.96%, 92.43%, and 91.76%, respectively. The cross-validation accuracy was 87.4027%. With the GA-SVM model, the identification accuracy for each oolong tea plant cultivar was above 80%. Results of oolong tea cultivars’ identification by the GA-SVM model are shown in the **Figure 3**.

**Figure 4:**
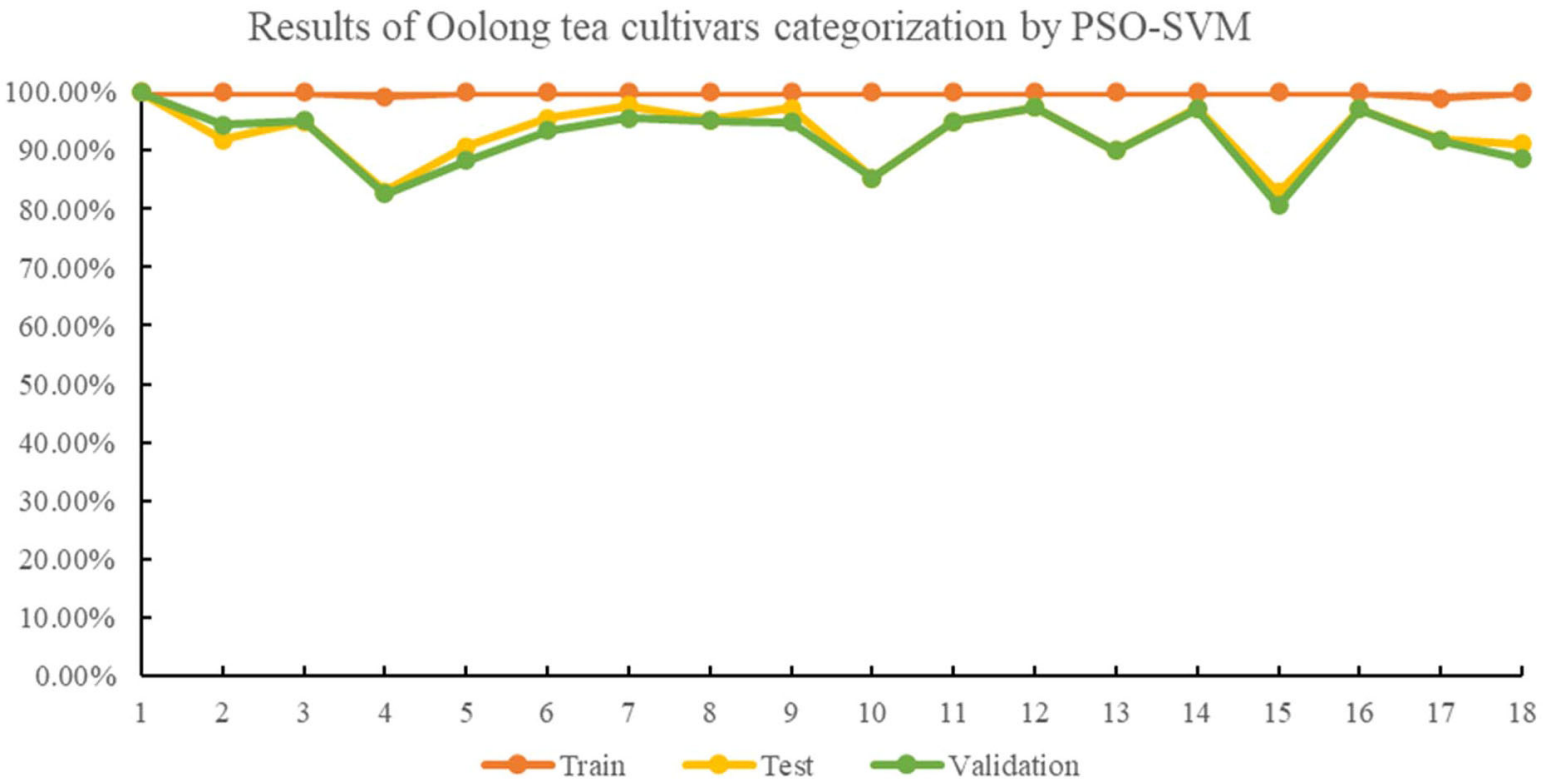
Results of oolong tea cultivar categorization by PSO-SVM.

In the PSO-SVM, the average accuracies for the training, testing, and validation sets were 99.91%, 93.05%, and 92.37%, respectively, with best c and g values of 20.7432 and 8.0080. The identification accuracies for the Ruixiang, Benshan, and Foshou cultivars in the testing and validation sets were improved compared to the GA-SVM model. The detailed results of each cultivar identification rate by PSO-SVM are shown in **Figure 4**.

**Figure 5:**
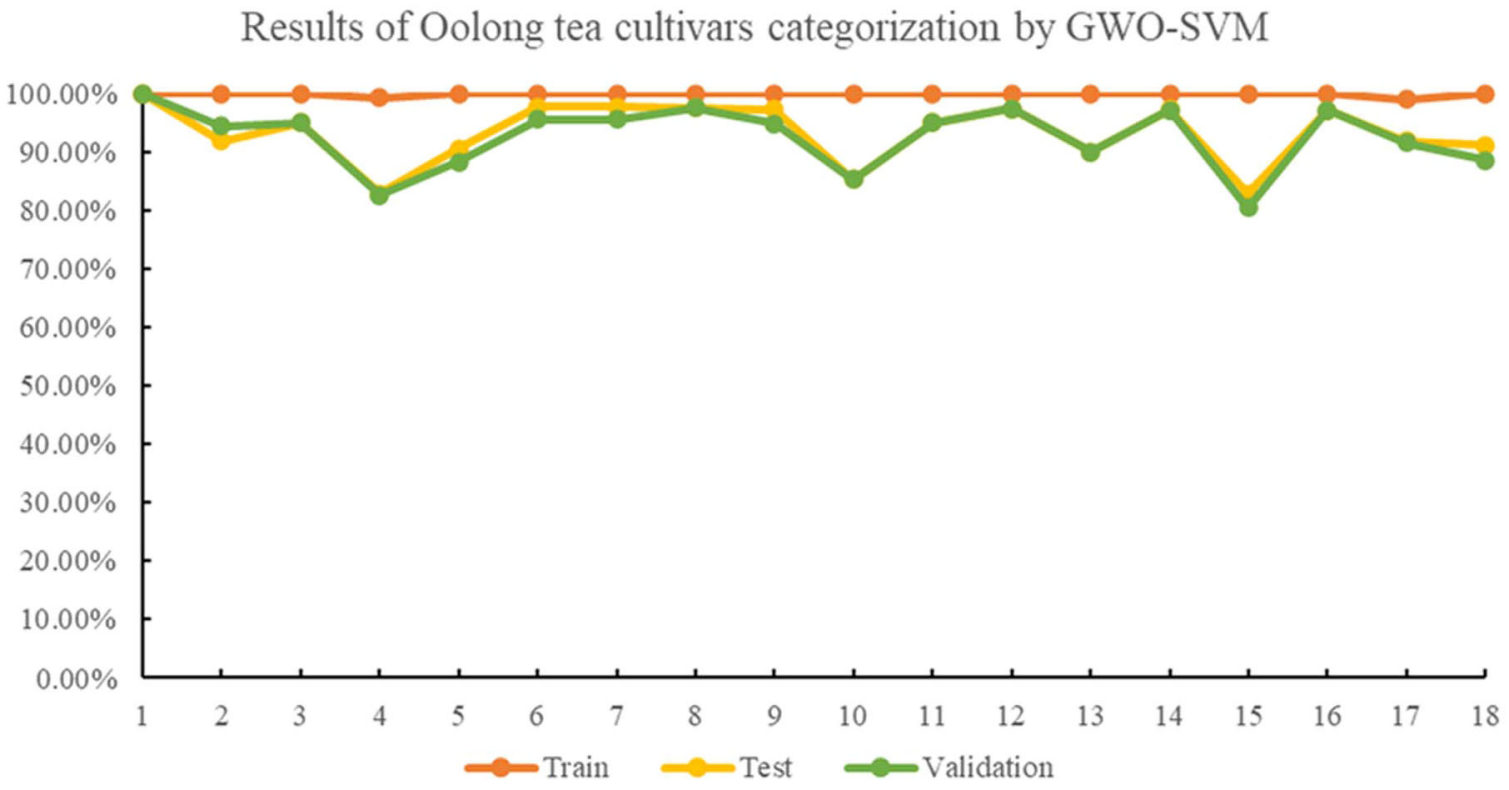
Results of oolong tea cultivar categorization by GWO-SVM.

Lastly, when the GWO algorithm was used to optimize the SVM classification model for identifying oolong tea cultivars, the average accuracies were higher than the PSO-SVM model, with 99.91%, 93.30%, and 92.63% for the training, testing, and validation sets, respectively. In the GWO-SVM, the best c and g values were 23.7723 and 7.4001, respectively. The identification rates for Shuixian, Ruixiang, and Dahongpao cultivars in the testing and validation sets further improved compared to the PSO-SVM model. However, the identification rates of other cultivars remained the same as the PSO-SVM model. **Figure 5** displays the identification results of 18 oolong tea cultivars by GWO-SVM. GWO, based on gray wolf behavior, has advantages over GA and PSO in optimizing SVM models. GWO mimics the behavior of gray wolves, which allows for better exploration and utilization of the search space. The algorithm’s simplicity in parameter settings and fast convergence rate also contribute to its potential superiority. Therefore, SVM recognition results optimized by GWO are better than GA and PSO in this study.

### 3.3 Germination period classification of 18 oolong tea cultivars

The 18 selected oolong tea cultivars can be classified into three types, early, mesophytic and late species. Additionally, 27 indicators have been selected for evaluating oolong tea cultivars. The average values of these indicators are used as characteristics for each cultivar. Color, texture, and spectral behaviors of the oolong tea cultivars vary among three germination periods, and Fisher discriminant analysis was used to evaluate the germination period of the cultivars. The mean value of 27 indicators of 18 oolong tea cultivars as input was Fisher discriminated, and the discriminant functions were as follows:


(3) $$\begin{array}{l} {\text{Y}}_{1}=-4.876*\text{h}+9.114*\text{s}-18.636*\text{r}+12.217*\text{b}-3.143*\\ \text{L}-5.471*\text{Asm}-3.089*\text{Var}-16.540*\text{Hom}+14.565*\text{Dis}-\\ 2.819*\sigma +5.969*\text{S}+15.693*\text{G}+0.101*\text{RVI}-0.244*\text{DVI}+3.937*\text{VOG} \end{array}$$

(4) $$\begin{array}{l} {\text{Y}}_{2\;=}3.941*\text{h}+7.469*\text{s}+12.911*\text{r}-8.468*\text{b}-2.090*\text{L}-4.088*\\ \text{Asm}-3.011*\text{Var}+7.450*\text{Hom}-5.551*\text{Dis}+4.832*\sigma -3.818*\text{S}-\\ 1.803*\text{G}+3.083*\text{RVI}-6.684*\text{DVI}+6.749*\text{VOG} \end{array}$$

**Figure 6:**
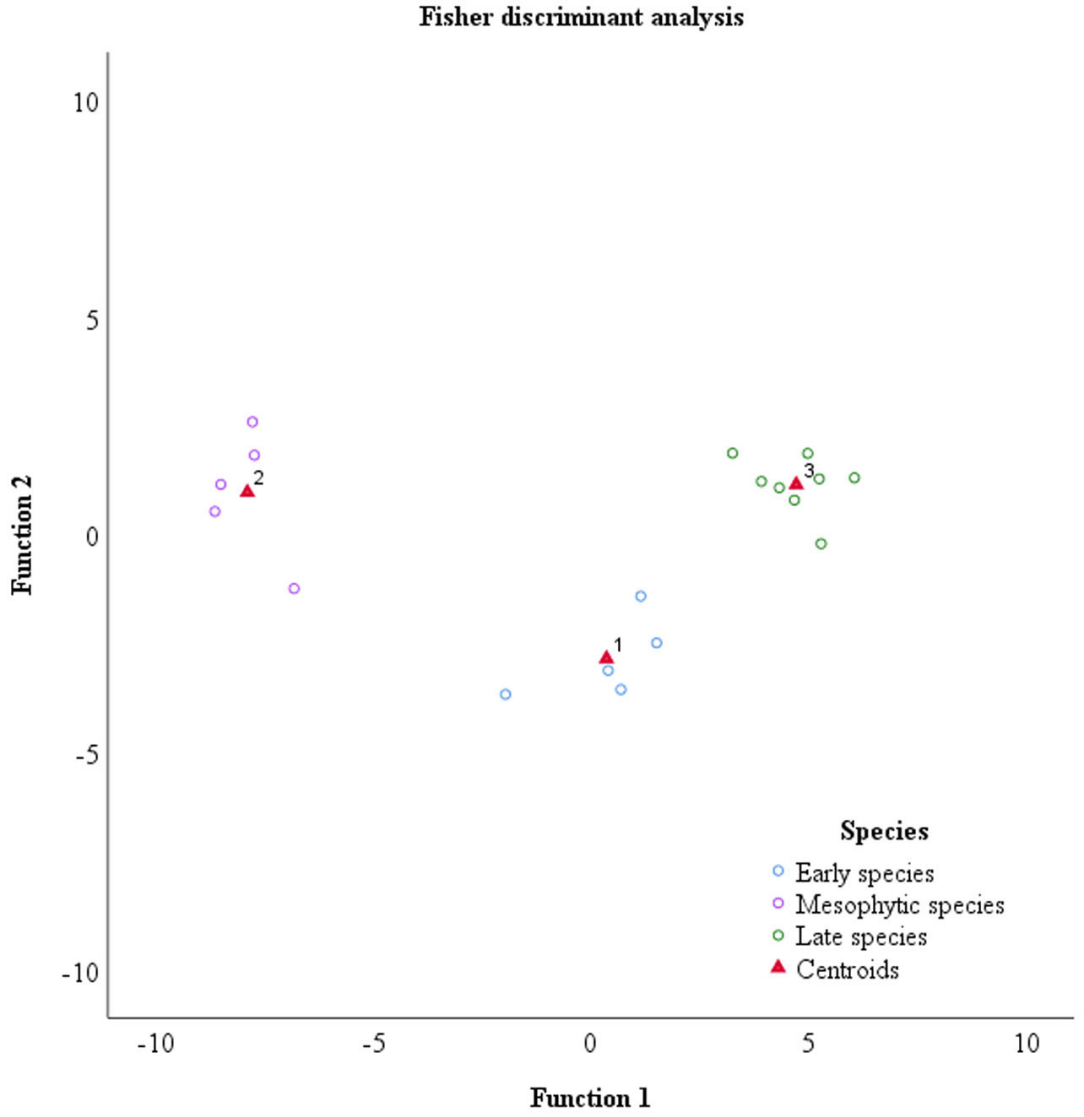
Discriminant analysis of germination period of oolong tea cultivars.

The class mean projection matrix represents the class center position of the three species. As can be seen in **Table 4**, tea cultivars of early, mesophytic and late species were correctly identified. The scatterplot, generated by plotting the two discriminant scores of three species, as shown in **Figure 6**, indicates a significant separation effect among different cultivars. This suggests that indicators (h, s, r, b, L, Asm, Var, Hom, Dis, σ, S, G, RVI, DVI, VOG) distinguishes the early, mesophytic and late species based on Fisher discriminant analysis.

**Table 4:** Discriminant analysis of the corresponding confusion matrix and projection matrix.

Species	Confusion matrix			Class mean projection matrix	
From/To	Early species	Mesophytic species	Late species	Function 1	Function 2
Early species	5	0	0	0.353	-2.845
Mesophytic species	0	5	0	-7.907	0.984
Late species	0	0	8	4.721	1.163

## 4 Discussion

### 4.1 Effects of different scales of datasets on oolong tea cultivar identification

**Figure 7:**
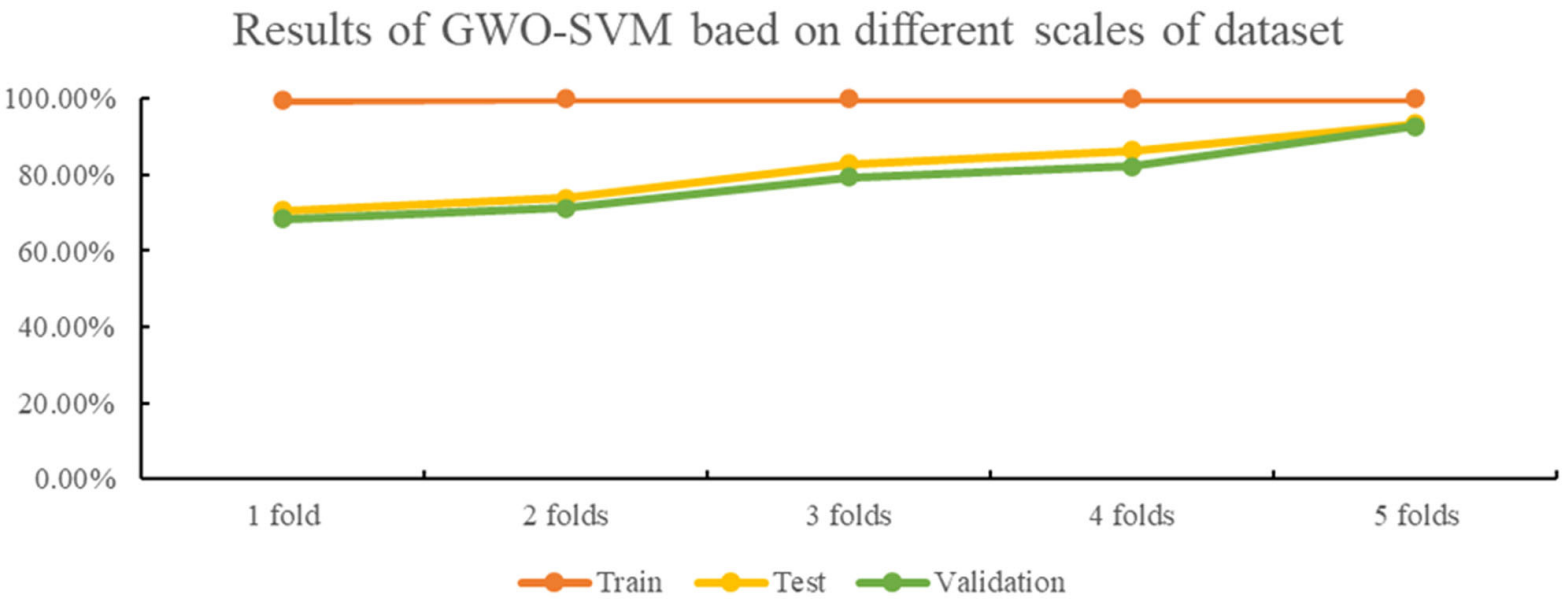
The results of GWO-SVM based on different scales of dataset.

The identification accuracies of the 18 different oolong tea cultivars were found to be above 80% with GA-, PSO-, and GWO-optimized SVM classification models, as seen in **Figures 3**–**5**. The GWO-SVM achieved high accuracies for both average identification rate and each species, with the identification rates of Shuixian, Baxian, Rougui, Dahongpao, Tieluohan, Benshan, Maoxie, Huangyan, Dangui, and Jinmudan improving in both test and validation sets. This research involved capturing and analyzing 3639 sets of images to explore how dataset size affects identification accuracy. The 18 oolong tea cultivars dataset was randomly divided into five parts (1/5, 2/5, 3/5, 4/5, and 5/5), and each dataset was then used for GWO-SVM modeling. These datasets were further divided into training, test, and validation sets (at a ratio of 3:1:1). As shown in **Figure 7**, the accuracies in the test and validation sets were less than 80% when the dataset was only 1, 2, or 3 folds. However, the accuracies of the test and validation sets improved with the increase in data scales, while the identification rate of the training set was not significantly affected by the scale of the dataset.

**Figure 8:**
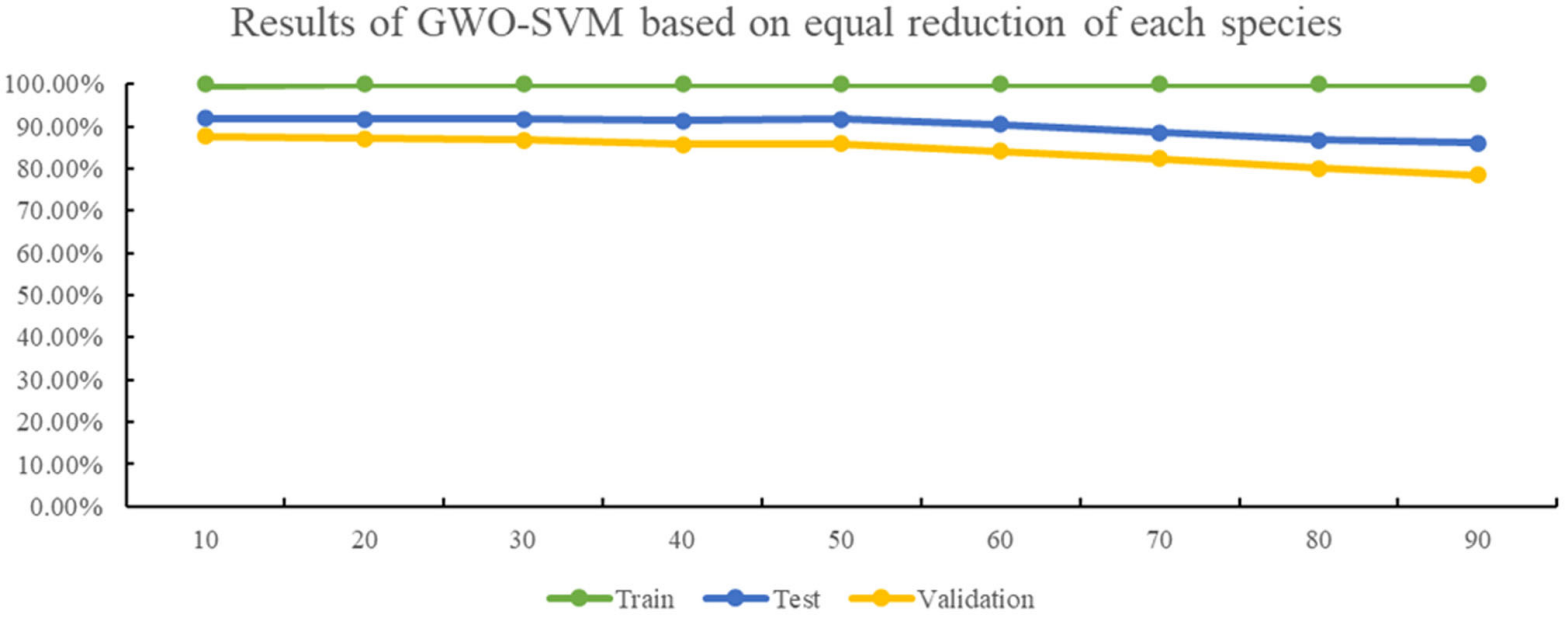
The results of GWO-SVM based on equal reduction of each cultivar.

Furthermore, the number of datasets used for each tea variety was reduced by the same amount, and the GWO-SVM was employed to create a tea cultivar recognition model. **Figure 8** illustrates that 10 sets of images were randomly selected and reduced for each variety during each trial. Despite minimal changes in the accuracy of the training set, the recognition accuracy of the test and validation sets decreased as the dataset size decreased. When the number of images obtained from each oolong tea cultivar decreased by 60 sets, the recognition accuracy of the model decreased significantly, with the accuracy of the validation set falling below 85%. Similarly, when 90 data sets of each oolong tea cultivar were reduced, the accuracy of the verification set decreased to less than 80%.

### 4.2 Variable importance analysis of selected indicators

**Figure 9:**
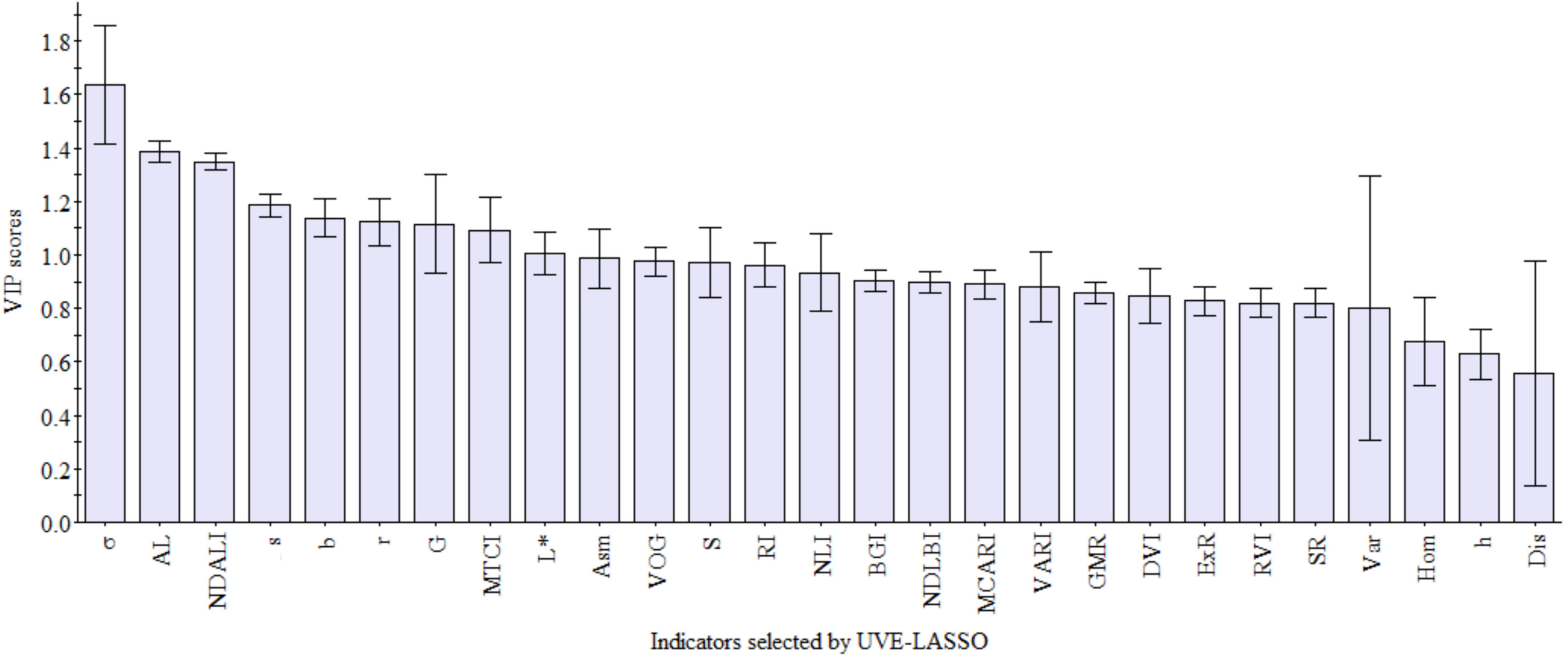
VIP scores plot of indicators selected by UVE-LASSO.

This study used UVE-LASSO to select 27 indicators that can be used for oolong tea cultivar identification. To investigate the contribution of these selected indicators, the variable importance in the projection algorithm was employed. **Figure 9** shows that all 27 factors are significantly important, with VIP scores ≥ 0.5. Among these, σ, AL, NDALI, s, b, r, G, MTCI, and L contributed the most (VIP >1.0). Compared to Cao (Cao et al., 2022b), it is evident that σ plays a crucial role in identifying tea plant varieties, including oolong tea cultivars. Additionally, color information plays a more important role in the classification of oolong tea cultivars than other tea plant cultivars.

### 4.3 Analysis of factors influencing the discriminant results

**Figure 10:**
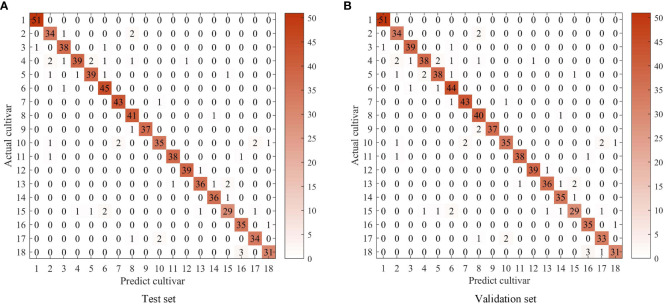
Confusion matrix of predict cultivar and actual cultivar in test and validation sets.

The Baijiguan, Shuixian, Yellow Rose, and Benshan differ greatly in color. Additionally, the texture information of 18 oolong tea cultivars is distinct. The spectral information of five bands is mainly related to quality components such as moisture, polyphenols, chlorophyll, and amino acids. This information varies between different oolong tea cultivars, providing the basis for tea plant identification using a multispectral imaging system. The confusion matrix of the test and validation sets is shown in **Figure 10**. It can be observed that Baxian was sometimes misjudged as Dahongpao in both the test and validation sets, likely due to their similar color. **Figure 9** also indicates that color characteristics play an important role in oolong tea cultivar classification. Jiulongpao cultivar was wrongly discriminated as Baxian, Rougui, Qilan, Ruixiang, Dahongpao, and Tieguanyin. The majority of wrongly discriminated cultivars were late species. Qilan was consistently discriminated as Baxian, Jiulongpao, Ruixiang, and Huangyan in both the test and validation sets. Ruixiang was misjudged as Qilan and Rougui. Tieluohan was misclassified as Yellow Rose and Dangui, likely due to their similar leaf texture and phenotypic appearance. Benshan was wrongly identified as Huangyan, likely due to their similar canopy structure and appearance. Jinmudan was wrongly discriminated as Foshou, possibly due to the similar texture and appearance of their leaves. Overall, the optimization of the GWO-SVM model improved the recognition rate of oolong tea cultivars. The factors influencing misjudgment mainly included similar canopy structure, leaf color and texture, and germination period of tea plants. Oolong tea cultivars with the same germination period can be misjudged. This model has demonstrated a high recognition rate specifically for the 18 oolong tea varieties. For other unmentioned cultivars, it is essential to collect data, optimize the model, and readjust the parameters accordingly. Moreover, when expanding the dataset, it is necessary to consider the variations in traits of tea varieties cultivated in different seasons and ecological environments across various regions.

## 5 Conclusion

Both spectral analysis and machine learning methods are valuable for processing multispectral data in tea classification and are effective in extracting useful information. This paper proposes, for the first time, the combination of UVE and LASSO to select proper multispectral indicators for tea cultivar identification and evaluation. The selected 27 factors combined with the GWO-optimized SVM classification model showed good identification. Tea cultivar recognition with computer technology, instead of traditional manual identification, is an inevitable attempt. Furthermore, based on the multispectral information, the Fisher discriminate analysis completely divided these oolong tea cultivars into early, mesophytic, and late species, indicating the feasibility of oolong tea cultivar germination classification by effective multispectral information. The rapid and accurate classification and identification of tea cultivars and germination period have practical implications across various areas of the tea industry, including variety conservation and management, tea quality control, market competitiveness, fraud prevention, and scientific research and genetic resource conservation.

## Data availability statement

The original contributions presented in the study are included in the article/supplementary material. Further inquiries can be directed to the corresponding authors.

## Author contributions

QC and GY: Conceptualization. BB, JC, LC, and FW: Resources, data curation. QC and BX: Methodology. QC: Writing—original draft preparation. GY and XM: Writing—review and editing. GY and PJ: Supervision. CZ and DD: Funding acquisition. All authors contributed to the article and approved the submitted version.
